# Oxidative DNA Damage and Arterial Hypertension in Light of Current ESC Guidelines

**DOI:** 10.3390/ijms252312557

**Published:** 2024-11-22

**Authors:** Radka Hazuková, Zdeněk Zadák, Miloslav Pleskot, Petr Zdráhal, Martin Pumprla, Miloš Táborský

**Affiliations:** 1 Department of Internal Medicine I-Cardiology, University Hospital Olomouc and Faculty of Medicine and Dentistry, Palacky University Olomouc, 77900 Olomouc, Czech Republic; akdar.hazukova@seznam.cz; 2 Department of Cardiology and Internal Medicine, Profi-Kardio, s.r.o., 50801 Hořice, Czech Republic; 3 IIIrd Department of Internal Medicine-Gerontology and Metabolism, Medical Faculty in Hradec Králové, University Hospital Hradec Králové, Charles University Prague, 50003 Hradec Králové, Czech Republic

**Keywords:** ESC guidelines, oxidative stress, concept on genesis, hypertension, PARP inhibitors, DDR, DNA damage and strand breaks, heart failure, cardiovascular disease, risk factors and therapy, γH2AX

## Abstract

A new insight into oxidative stress is based on oxidative deoxyribonucleic acid (DNA) damage. DNA is the pivotal biopolymer for life and health. Arterial hypertension (HT) is a globally common disease and a major risk factor for numerous cardiovascular (CV) conditions and non-cardiac complications, making it a significant health and socio-economic problem. The aetiology of HT is multifactorial. Oxidative stress is the main driver. Oxidative DNA damage (oxidised guanosine (8OHdG), strand breaks (SSBs, DSBs)) seems to be the crucial and initiating causal molecular mechanism leading to HT, acting through oxidative stress and the resulting consequences (inflammation, fibrosis, vascular remodelling, stiffness, thickness, and endothelial dysfunction). In light of the current European Society of Cardiology (ESC) guidelines with defined gaps in the evidence, this manuscript, for the first time, (1) summarizes evidence for oxidative DNA damage in HT and other CV risk factors, (2) incorporates them into the context of known mechanisms in HT genesis, (3) proposes the existing concept of HT genesis innovatively supplemented with oxidative DNA damage, and (4) mentions consequences such as promising new targets for the treatment of HT (DNA damage response (DDR) pathways).

## 1. Introduction

Systemic arterial hypertension (HT) is a widespread chronic cardiovascular (CV) disease which affects about 30% of adults [1,2]. The occurrence of HT rises with age [1,2]. The majority of HT cases, approximately 90%, are termed “essential” because the exact cause remains unknown [1,2]. Importantly, HT also represents one of the CV risk factors, of which some are modifiable (HT, dyslipidaemia, cigarette smoking, sedentary lifestyle, hyperglycaemia) and others are not modifiable (age, male gender, family history) [1,2,3,4,5,6,7,8,9]. HT as a risk factor, acting via vascular-wall- remodelling (metabolic, functional, structural) and haemodynamic consequences, mediates CV diseases of atherosclerotic or non-atherosclerotic origin (coronary artery disease, atrial fibrillation, heart failure, peripheral arterial and aortic diseases, etc.) [1,2,3,4,5,6]. Despite advances in diagnosis, therapeutics, and the promotion of a healthy lifestyle, HT remains one of the leading causes of morbidity, disability, and mortality [1,2]. Thus, HT represents a serious health problem.

The aetiology of HT is based on multiple factors and their complex interactions [1,2]. Oxidative stress (ox-stress) is widely accepted as the crucial and common driver in the genesis of CV diseases including HT [1]. Exogenous and endogenous CV risk factors as well as redox factors alter the physiological redox homeostasis toward oxidant dominance (reactive oxygen species (ROS), reactive nitrogenous species (RNS)) [1,2,10]. Exogenous redox factors include cigarette smoking, ultraviolet in sunlight, and irradiation. Endogenous redox factors consist of metabolic processes and attenuation of antioxidants (through genetic polymorphisms, epigenetic or posttranslational protein modification that may alter enzymes, signalling molecules, transporters, amplifiers, effectors, or sensors). This redox imbalance where oxidants outweigh antioxidants is called ox-stress [11]. Ox-stress with ROS and RNS, together with their complex consequences (proliferative, proinflammatory, profibrotic) has been postulated to be the basic driver in the aetiology of vasculopathy, hypertension, and other CV diseases [1]. HT vasculopathy is characterised by endothelial dysfunction, higher endothelial permeability for both inflammatory cells and low-density lipoprotein (LDL), vascular wall remodelling (hypertrophy, inflammation, fibrosis, thickness, stiffness, and sometimes atherosclerosis), and geometric rearrangement of vascular networks [1,3,12,13,14,15,16,17,18,19,20,21,22].

**Figure 1:**
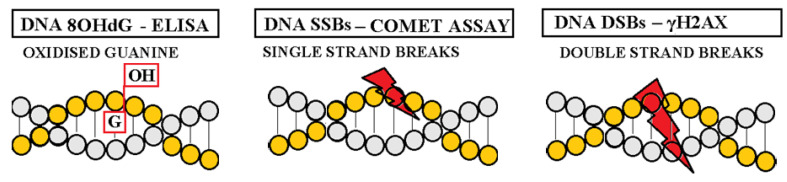
DNA damage types. **Legend, abbreviations**. The most serious DNA damage types from less serious to the most serious (8-hydroxy-2′deoxyguanosine (8OHdG), single strand breaks (SSBs), double strand breaks (DSBs)). ELISA, enzyme-linked immunosorbent assay; γH2AX, phosphorylated histone H2AX. DNA, deoxyribonucleic acid. *Copyright is not needed, all illustrations are originally created by the author of this text based on known facts*.

**Figure 2:**
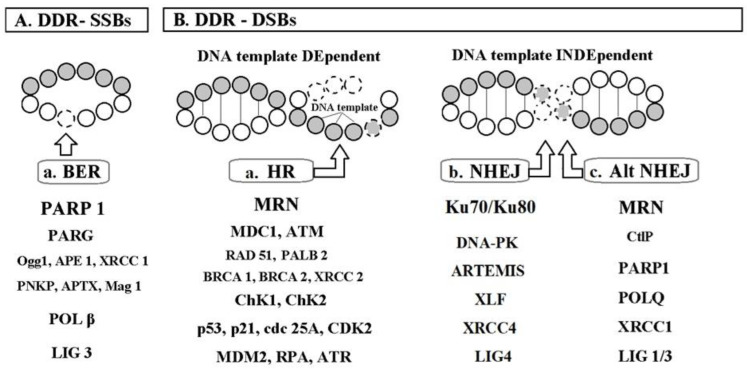
Deoxyribonucleic acid (DNA) damage response (DDR) system for (**A**) oxidised bases and single strand breaks (SSBs); (**B**) double strand breaks (DSBs). Legend, abbreviations. A. DDR—SSBs, deoxyribonucleic acid (DNA) damage response (DDR) system for DNA single strand breaks (SSBs) and base damage. A.a. The BER (base excision repair) pathway is composed of the following: PARP, Polymeric Adenosine diphosphate Ribose Polymerase; PARG, Polymeric Adenosine diphosphate Ribose Glycohydrolase; Ogg1, 8-oxoguanine (8-oxoG) DNA glycosylase 1; APE1, apurinic endonuclease 1; XRCC1, X-ray repair cross-complementing protein 1; PNKP, Polynucleotide kinase phosphatase; APTX, Aprataxin; Mag 1, 3-methyl-adenine DNA glycosylase; POL β, DNA polymerase β; LIG 3, DNA ligase 3. (**B**). DDR—DSBs, deoxyribonucleic acid (DNA) damage response (DDR) system for DNA double strand breaks (DSBs). B.a. HR (homologous recombination) pathway—a relatively slow process in the S/G2 phases, requiring a DNA template (a 3′ single-stranded DNA (ssDNA) overhang = the 3′ DNA tail); MRN complex, a complex of MRE 11, RAD 50, and Nijmegen breakage syndrome 1 protein (NBS1); MDC1, mediator of DNA damage checkpoint 1; ATM, protein kinase ataxia-telangiectasia mutated (serine/threonine protein kinase ATM); PALB, Partner And Localizer of BRCA2; BRCA, Breast cancer protein; XRCC2, X-ray repair cross-complementing protein 2; ChK1, Checkpoint Kinase 1 (transducer kinase for ATR); ChK2, Checkpoint Kinase 2 (transducer kinase for ATM), p53, tumor suppressor protein 53; p21, tumor suppressor protein 21; cdc 25A, cell division cycle 25A phosphatase; CDK, Cyclin dependent kinase; MDM2, Murine double minute 2; RPA, replication protein A; ATR, ataxia telangiectasia and Rad3-related protein kinase (serine/threonine protein kinase ATR). B.b. NHEJ (non-homologous end joining)—an error prone pathway, but a fast, cell-cycle-independent process. Ku70/Ku/80, heterodimer recognising a DNA DSB motif; DNA-PK, DNA-dependent protein kinase; Artemis, a protein phosphorylated by DNA-PK; XLF, XRCC4-like factor; XRCC4, X-Ray Repair Cross Complementing protein 4 stabilizes LIG 4; LIG4, DNA ligase 4. B.c. Alt NHEJ (alternative pathway for NHEJ); CtIP, carboxy-terminal binding protein (CtBP) interacting protein; POLQ, POL *Q* gene-encoded DNA polymerase theta [28,32,33]. *Copyright is not needed, all illustrations are originally created by the author of this text based on known facts*.

Thanks to advances in research on sub-cellular levels, oxidative deoxyribonucleic acid (ox-DNA) damage (8-hydroxy-2’-deoxyguanosine (8OHdG), single strand breaks (SSBs), double strand breaks (DSBs)) has been recognized and discussed in the context of ox-stress and simultaneously in the context of new therapeutic targets [23,24,25,26,27,28,29] (Figure 1). DNA damage response (DDR) substances (e.g., polymeric adenosine-diphosphate ribose polymerase (PARP)) seem to be promising causal targets for innovation in CV pharmacotherapy [28,30,31,32,33,34,35,36,37,38,39] (Figure 2).

In light of the current European Society of Cardiology (ESC) guidelines with defined gaps in the evidence (“The drivers of worsening blood pressure control”) [1], this review tries at least partially to fill this gap by revealing the possible basic driver of HT genesis. Thus, the aims of this review were (1) to summarize the evidence for ox-DNA damage in HT and other risk factors, (2) to put the summarized evidence into the context of known mechanisms of HT genesis, (3) to propose a concept of HT genesis innovatively supplemented with ox-DNA damage, and (4) to mention consequences such as promising new targets for the treatment of HT (DDR substances, Figure 2).

## 2. DNA, ox-DNA Damage

### 2.1. DNA

DNA is the central biopolymer of life [40] and is crucial for the functional and structural integrity of the human organism [40]. The majority of DNA, called “genomic”, is located in the cell nucleus [40]. A minor amount is located in mitochondria, where it encodes enzymes involved in oxidative phosphorylation, the main energetic process in cells [40].

### 2.2. DNA Damage

DNA is very sensitive to destruction caused by the exogenous or endogenous environment [28,40]. Each day, there are around 10^4^ instances of DNA damage [28]. Several types of DNA damage exist: base damage (8OHdG), strand breaks (SSBs, DSBs), sugar damage, cross-links, and clustered damaged sites [24,25,26,27,28]. The three most significant types of DNA damage are 8OHdG, SSBs, and DSBs, from least serious to most serious [24,25,26,27,32,33]. The most frequent are SSBs [28,32,33]. The most deleterious DNA lesions are DSBs, but they are significantly less frequent than SSBs [24,25,26,27,28]. Numerous studies have illustrated that DNA lesions are caused by ROS arising directly or indirectly from exposure to both exogenous (cigarette smoking, ultraviolet light, ionizing radiation, heavy metals) and endogenous factors (cell metabolism, DNA replication errors, etc.) [11,28]. In this review, we have focused on the most serious types (8OHdG, SSBs, DSBs) of nuclear (not mitochondrial) DNA damage and on the relevant biomarkers or analytical methods in the relevant order (enzyme-linked immunosorbent assay (ELISA), alkaline comet assay (comet) and phosphorylated histone H2AX (γH2AX)) in HT and other CV risk factors [24,25,26,27].

## 3. ox-DNA Damage—CV Risk Factors

### 3.1. Hypertension

#### 3.1.1. Humans

In our previous paper (a systematic review with meta-analysis of precisely selected controlled studies on hypertensive humans and severe ox-DNA damage), several main findings were recognized for the first time [26]. These findings must be briefly reiterated here:

##### Increased ox-DNA Damage in Isolated HT

**Figure 3:**
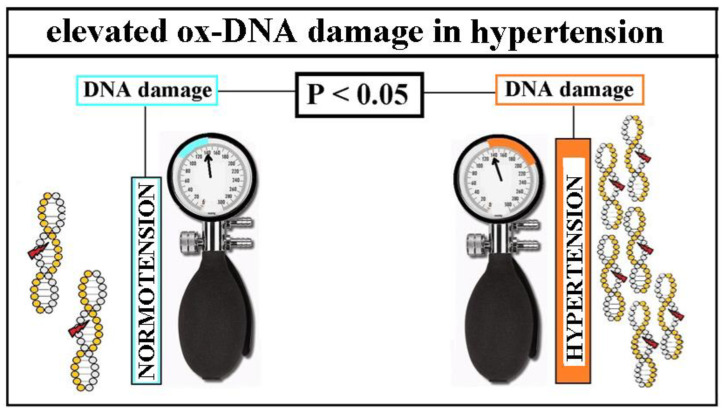
DNA damage is increased in hypertension (in humans, animals, and cell cultures). **Legend, abbreviations**. ox-DNA damage, oxidative DNA damage; DNA, deoxyribonucleic acid; blue color, normal blood pressure; orange color, arterial hypertension; statistical significance *p* < 0.05. *Copyright is not needed, all illustrations are originally created by the author of this text based on known facts*.

The first main innovative finding was that ox-DNA damage was consistently increased in people with hypertension (*n* = 843 patients (pts)) compared with healthy controls (*n* = 705) [26] (Figure 3). Unfortunately, only the lesser forms of ox-DNA damage (8OHdG, SSBs/comet) were tested in these studies: (a) 8OHdG in blood (*n* = 122 pts), (b) 8OHdG in urine (*n* = 326 pts), and (c) SSBs/comet in blood (*n* = 395 pts) [26].

There were no studies on the most severe ox-DNA damage (DSBs/γH2AX) in humans with hypertension [26]. Regarding HT types, essential, gestational, and white-coat HT (WCH) were tested in humans. In accordance with assumptions, DNA damage in WCH was comparable to that in healthy controls [26].

##### Increased ox-DNA Damage in HT with Additional Adverse Characteristics

The second original finding was that greater DNA damage (8OHdG in blood/urine; SSBs/comet assay in blood) was observed in HT cases with additional adverse characteristics (concentric cardiac hypertrophy, coronary artery disease, old age, non-dipper status, sustained/untreated HT, etc.) than in cases of simple HT alone [26].

##### Mean Differences in ox-DNA Damage

The third interesting de novo finding was that the mean difference between hypertensives and matched healthy controls, measured in SSBs detected by the comet assay in peripheral blood cells/lymphocytes (PBCs/Ly), was 14.7 (6.4; 23.0) arbitrary units (A.U.) [26]. Using 8OHdG/ELISA in urine, the mean difference between hypertensives and matched healthy controls was 7.5 (6.6; 8.6) ng.mg^−1^ creatinine [26].

##### Correlation with ox-DNA Damage

The fourth interesting original finding was related to DNA damage correlation [26]. Among the tested parameters (cholesterol, LDL, age, blood pressure (BP), total antioxidant status (TAS), plasma fasting glucose (glc)) for which the correlation with both DNA damage types (8OHdG; SSBs/comet assay) was tested, only the total antioxidant status (TAS) showed a consistently stronger negative significant correlation for both methods (8OHdG; urine; *n* = 105 pts); (SSBs/comet, blood; *n* = 265 pts); (r = −0.670 to −0.933; *p* < 0.05) [26]. Surprisingly, regarding blood pressure (BP), the correlation was found in humans only for SSBs detected by the comet assay in blood and not for 8OHdG [26]. This finding supports our presumption that 8OHdG is not a suitable marker in cardiology, at least in the context of HT [26]. Despite that, we believe that the findings relating to 8OHdG (urine) were seen for serum glycosylated haemoglobin (HbA1c; r = 0.670; *p* < 0.05) [26]. Other parameters considered in these studies displayed weaker significant correlation (*p* < 0.05) with DNA damage (8OHdG), namely, high-sensitivity C-reactive protein (hs CRP) (r = 0.315), brachial–ankle pulse wave velocity (PWV) (r = 0.330), ischaemia-modified albumin (IMA) (r = 0.396 to 0.400), pro-oxidant/antioxidant balance (PAB) (r = 0.372), protein carbonyl (PCO) (r = 0.370 to 0.243), advanced oxidation protein products (AOPPs) (r = 0.411), extent of coronary heart disease (CHD) (r = 0.232 to 0.424), and total thiol (T-SH) (r = −0.290 to −0.410) [26]. Other parameters did not exhibit significant correlation results (*p* ≥ 0.05) [26].

#### 3.1.2. Animals and Cell Cultures

Importantly, these aforementioned findings in humans, i.e., that severe ox-DNA damage is consistently higher in people with HT than in matched normotensive people, has recently been confirmed in both animals and cell culture through controlled and carefully selected studies under strict experimental conditions [27]. The authors believe that strictly defined laboratory conditions may exclude the misinterpretation of the results [27].

At this time, we will briefly review the included studies with animal HT models and DNA damage. All three types of ox-DNA damage (8OHdG, SSBs, DSBs) were tested in animals [27]. Regarding the number of subjects, there were, after subtracting possible duplicates, *n* = 86 HT animals. The mean age of HT animals was 22.3 ± 19.0 weeks. The number of animal normotensive controls was *n* = 74 after subtracting possible duplicates [27].

Among animals, various types of HT models were used: (1) genetically determined (spontaneously hypertensive rats (SHRs)), (2) humorally induced (angiotensin II (Ang II), aldosterone (Ald), deoxycorticosterone acetate (DOCA)), using volume overload-induction, (4) surgically induced (2-kidney 1-clip (2K1C), aortic banding), (5) using diet-induction (sodium chloride (NaCl)) [27]. All the studies reported higher DNA damage levels in hypertensive animals [27]. Thus, animal models have supported the results in humans [26,27].

Similarly, we briefly mention results from carefully selected controlled studies on cell cultures. In all selected studies, a higher level of DNA damage was seen compared with controls. All three types of ox-DNA damage (8OHdG, SSBs, DSBs) were tested in cell cultures. In cell cultures, the HT model was induced humorally using Ang II or Ald [27].

### 3.2. Cigarette Smoking

To the best of our knowledge, the data on cigarette smoking and severe ox-DNA damage (8OHdG, SSBs/comet, DSBs/γH2AX) seem to be clear regarding acute smoking exposure and human studies (see below) [41,42,43,44,45,46].

On the other hand, in the case of chronic cigarette smoking, the data seem to be slightly unclear, with the possibility of artifacts (see below). However, in order to reduce misinterpretation and confounding factors, we underline the importance of strictly laboratory conditions [47,48].

If we approach this issue in detail, available human data consistently demonstrate higher ox-DNA damage after acute cigarette smoking exposure (8OHdG [41], SSBs/comet [42,43,44], DSBs/γH2AX [45,46]). Surprisingly, in the case of chronic cigarette smoking, the results on ox-DNA damage in human studies are not clear. It has been shown that the combination of chronic and acute cigarette smoking increases ox-DNA damage levels (8OHdG) [41], (SSBs/comet) [43]. These results should be interpreted with caution, because results may be altered by acute exposure [41,42,43]. Another two studies in humans centred on the effect of chronic cigarette exposure on ox-DNA damage are controversial. A study by Ganapathy did not find changes (8OHdG) [41]. However, by contrast, Yao found higher DNA damage (8OHdG, urine) in chronic smokers than in non-smokers, assessed as the mean value of DNA damage (31.4 ± 18.9 versus 14.4 ± 7.6 nM; *p* = 0.0004; 23.5 ± 21.3 versus 12.6 ± 13.2 μg.g^−1^ creatinine; *p* = 0.028) [49]. Notwithstanding, at this point, it has to be underlined that both animal studies consistently documented higher ox-DNA damage linked to chronic cigarette smoking exposure in strict experimental conditions (8OHdG [47], 8OHdG, DSBs/γH2AX [48]).

Special attention should be paid to a study by Aoshiba 2012 [48]. This human study did not find any changes in ox-DNA damage (8OHdG, DSBs/γH2AX) 3 months after stopping chronic cigarette smoking [48].

However, a human study that detected a decrease in 8OHdG introduces confusion [50].

### 3.3. Age

The relationship between ox-DNA damage and age does not seem clear. There are some factors complicating relevant interpretation: (1) the great heterogeneity in DNA damage types, used methods, and organisms and (2) the small number and size of existing studies. Thus, it is impossible to draw serious conclusions, but it is necessary to be cautious and keep in mind the possible influence of age on DNA damage.

For 8OHdG/urine in humans, a higher level was observed in healthy elders (*n* = 30; mean age 69 years) than in younger healthy controls (*n* = 30; mean age 41 years) [51]. Similarly, a higher level of 8OHdG/urine was also found in older people with hypertension (*n* = 30, mean age 72 years) than in younger people with hypertension (*n* = 30, mean age 41 years) in the same study [51]. However, in this study results may be influenced by HT [26,51]. Similarly, older participants exhibited a higher level of DNA damage (8OHdG in pulmonary cells) than matched younger controls [52]. Although for SSBs/comet/blood in humans, no differences were found among healthy younger (*n* = 47; 20–34 years), older (*n* = 49; 60–74 years) or elderly healthy humans (*n* = 74; 90–98 years) [53], another human study found that older people with diabetes (*n* = 96; ≥38 years) had higher SSBs/comet/blood (PBCs) than younger people with diabetes (*n* = 23; 13 years) [54]. However, in this study results may be influenced by diabetes. Similarly, Wolf described higher SSBs/comet in pulmonary cells in older humans compared to younger humans [52].

In humans, higher DSBs/γH2AX was detected in the elderly in a very small group of patients with generally defined CV diseases [55]. This Lewis study requires careful interpretation of the DSBs/γH2AX finding because of additive CV diseases [55], although Ahuja found higher DSBs/γH2AX (cardiomyocytes from autopsy) in a healthy older man (64 years) than in a healthy younger man (30 years) [56].

By contrast, another study with unspecified CV diseases with left ventricular ejection fraction 40% showed no differences in DSBs/γH2AX (cardiomyocytes) between younger people (*n* = 10; 2–65 years) and older people (*n* = 15; 65–83 years) [57].

All selected animal studies showed higher DNA damage levels in older animals when compared with younger (SSBs/comet; DSBs/γH2AX), independently of health state [56,58,59,60].

### 3.4. Gender

In human studies, the results on DNA damage according to gender also remain inconclusive. In healthy humans, the majority of studies found no gender differences in DNA damage (8OHdG, SSBs/comet) in blood including peripheral blood cells (PBCs) [53,61,62,63,64].

For unknown reasons, men in two studies exhibited higher DNA damage levels (SSBs/comet/PBCs) than women matched in age—importantly, with no suspected link to a protective effect of premenopausal age [64,65]. This finding is partially supported by an animal model (SSBs/comet/in liver), although the less severe DNA damage biomarker (8OHdG) did not exhibit gender differences (liver) [66].

Completely contradictory data to the above findings linked to a gender impact on DNA damage are brought by both of two further studies, where higher values are observed in women [67,68]. Wu’s study, with the most enrolled individuals (10^3^), found greater DNA damage (8OHdG/urine) in females with defined normal ranges (*n* = 486; 43.9 ± 42.1 ng.mg^−1^ creatinine) when compared to males with defined normal ranges (*n* = 548; 29.6 ± 24.5 ng.mg^−1^ creatinine) [66]. A study by Moller also found higher DNA damage (SSBs/comet/PBCs) in women (*n* = 14) than in men (*n* = 7) [68].

Unfortunately, to the best of our knowledge, there are no results on DSBs/γH2AX relating to gender.

### 3.5. Exercise/Sedentary Lifestyle

In the context of CV disease, exercise in the range of submaximal intensity level (75–85% max heart rate (max HR)) is cardioprotective. By contrast, a sedentary lifestyle is one of the main modifiable CV risk factors. However, studies are based on exercise rather than lifestyle, as exercise is easier to define and studies on exercise are easier to grasp. However vigorous exercise (too long, or at excessively high intensity of 90–100% max HR) is out of the cardioprotective range and may be harmful.

In humans, it has been shown that dynamic acute exercise (30–90 min) at submaximal HR (75–85% max HR) does not change the level of DNA damage in blood (8OHdG, SSBs/comet), either in trained or untrained subjects (*n* = 317 in total) [69,70,71,72]. In contrast, acute dynamic vigorous exercise of high intensity (90–100% max HR) or of ultra-long duration increases DNA damage in blood in young subjects (*n* = 106; 8OHdG; SSBs/comet/PBCs) [73,74,75] as well as in urine (*n* = 24; 8OHdG) [76]. Application of force against heavy resistance independently of acute/chronic exercise increases blood 8OHdG [77,78]. Additionally, chronic heavy dynamic exercise increases blood 8OHdG [79], whilst chronic lower-level exercise does not [61,80]. Unfortunately, to the best of our knowledge, there are no studies on exercise and DSBs (γH2AX). This section will be discussed below in detail. 

In healthy younger human adults (middle-aged and young), acute running at submaximal HR (30–90 min, 4–21 km, 75–85% max HR) [69,70,71,72] does not induce DNA damage (SSBs/comet/blood) [69,70,71], (8OHdG/blood) [72]. In contrast, acute dynamic high intensity (90–95% max HR) or acute ultra-long exercise (3–150 h, 50–330 km) does increase DNA damage (8OHdG/blood) [73], (SSBs/comet/PBCs) [74,75], and (8OHdG/urine) [76]. Importantly, in cases of strength exertion against heavy resistance (60 min, 80% max HR), both acute and chronic activity increased blood 8OHdG [77,78,81].

Chronic exercise (6–10 months) in a heavy mode (10 h/week, *n* = 14 teenagers) increased DNA damage (8OHdG/blood) [79], whilst in a low mode (3 h/week, *n* = 96 young adults) it did not [61].

There are also other studies [82,83,84].

### 3.6. Diabetes Mellitus

In controlled human studies, there are quite consistent results with increased DNA damage in subjects with diabetes mellitus (*n* ≥ 437 people with diabetes in total). Similarly consistent results were seen in cases with other CV risk factors as mentioned above (hypertension, vigorous exercise, acute cigarette smoking). In people with diabetes, there were increases in several DNA damage markers (8OHdG in blood [85,86,87,88], 8OHdG in urine [67,89,90], and SSBs/comet/blood/PBCs [67,91,92,93]).

In only two human studies were no differences found in DNA damage (SSBs/comet/blood/PBCs), probably because the cohort was too small (*n* = 12 diabetics per study) [83,84].

Interestingly, increased DNA damage (8OHdG/plasma/urine) in humans was also observed in gestational diabetes mellitus (GDM) [85,86,87,88,89,90,91,92,93,94]. Urbaniak’s review summarises studies on GDM and DNA damage [94]. In Urbaniak’s review, one of the included studies found that 8OHdG concentrations of ≥8.01 ng.mg^−1^ creatinine can be probably a significant indicator of ox-stress and consequently of a higher risk of GDM development [94].

With respect to types of diabetes, type 2 diabetes mellitus (T2DM) (*n* = 52) showed higher DNA damage (SSBs/comet/blood/PBCs) in adults when compared to T1DM (*n* = 44) [54]. Lorenzi found higher DNA damage (SSBs/comet/blood/PBCs) in T1DM compared to controls [91].

The 8OHdG (serum) level was significantly greater in prediabetes (*n* = 33; 64.7 ± 10.4 years; 516.5 ± 260 pg.mL^−1^) compared to controls (*n*= 98; 66.2 ± 11.2 years; 177.8 ± 91 pg.mL^−1^; *p* < 0.01). The diabetes group (*n* = 35; 70 ± 8.4 years; 1926.9 ± 1197 pg.mL^−1^) had the highest level of 8OHdG, being approximately four times greater compared to the prediabetes group (*p* < 0.001) [87].

8OHdG in PBCs was higher in T2DM (*n* = 108) than controls (*n* = 65); (median ± interquartile range [IQR], 3.19 ± 2.17 versus 0.38 ± 1.00 ng.mL^−1^) and higher in T2DM with microangiopathy (*n* = 56) than without microangiopathy (*n* = 52) (median ± IQR, 3.34 ± 1.87 versus 2.71 ± 2.26 ng.mL^−1^) [88].

In addition, animal models showed higher DNA damage in diabetics (8OHdG, aortic cells), and similar results were found in cell lines (SSBs/comet/cardiomyocytes). In the case of DSBs/γH2AX, the single study with experimental mice found no differences in DNA damage (DSBs/γH2AX, nerve cells) [95].

Other papers did not test people with diabetes, and thus they are irrelevant and not discussed here ((a) sitagliptin suppressed oxidative stress/γH2AX/in chronic animal cerebral hypoperfusion, (b) with or without diabetic kidney disease, c/the extent of coronary artery lesions in elderly/ageing people with T2DM).

### 3.7. Dyslipidaemia/Hypercholesterolaemia

DNA damage (8OHdG) was analysed in blood (serum, plasma) and was higher in dyslipidaemia/hypercholesterolaemia/subjects with lower doses of statins when compared to relevant controls [96,97,98]. Similar data with increased DNA damage levels (cardiomyocytes, 8OHdG) were observed in animals with a high-fat diet [99]. In controlled human studies, the SSBs (comet/PBCs) were analysed, with increased levels in dyslipidaemia/apolipoprotein A5 allele (C-allele) [93,100,101,102,103,104]. To the best of our knowledge, data on DSBs/γH2AX in humans related to dyslipidaemia/hyperlipidaemia are not available despite the existence of data from human cell cultures [105,106]. Data on DSBs/γH2AX are available from animal models and animal cell cultures [107,108]. Lee evaluated the combination of diabetes and dyslipidaemia [108]. Further discovered studies are irrelevant.

### 3.8. Diet, Obesity

In humans (*n* ≥ 377 obese individuals in total), subjects with high visceral fat area/obese/prior bariatric surgery exhibited increased DNA damage (SSBs/comet/PBCs) compared with those with low visceral fat area/non-obese/after bariatric surgery [109,110,111,112,113,114,115,116,117]. On the other hand, in humans (total of *n* = 88 obese individuals), there were also non-significant results (SSBs/comet/PBCs) [118,119]. Caloric restriction and a high-carbohydrate, low-protein diet led to 30% weight loss, which was paralleled by decreased DNA damage (SSBs/comet) [102].

In animals, a high-fat diet induces DNA damage (8OHdG) [99]. Additionally, in animals, a high-fat diet induces an increase in DNA strand breaks, with a preventive effect of vitamin E [120]. In obese animals, Setayesh found increased DNA damage (8OHdG) and showed that gallic acid may be preventative [102,103,104].

## 4. The Concept of HT Genesis Innovatively Supplemented by ox-DNA Damage

**Figure 4:**
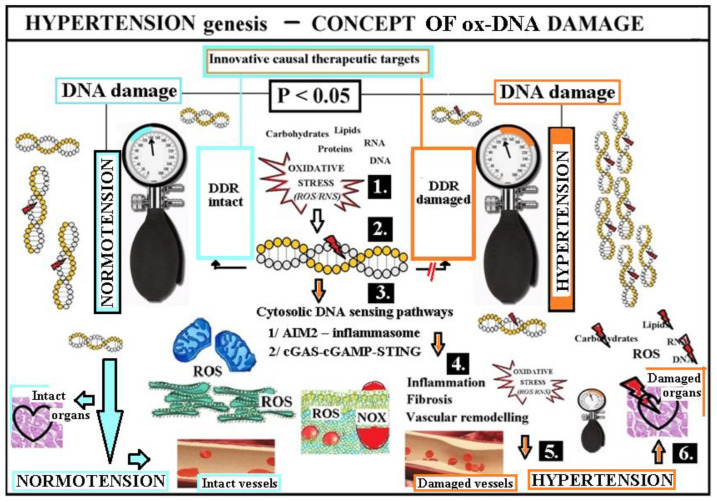
Hypertension genesis—the concept innovatively supplemented by ox-DNA damage. **Legend, abbreviations.** DNA is the crucial biopolymer for health and life [40]. The majority of DNA is located in the cell nucleus [40]. An uncontrolled imbalance between oxidants and antioxidants with a predominance of oxidants is known as oxidative stress [10,11,12,13]. Oxidative stress is driven by uncontrolled production of reactive oxygen species (ROS) or by an insufficiency of ROS scavengers (e.g., super-oxide-dismutase (SOD)) [10,11,12,13]. ROS arise from endogenous and exogenous sources (cigarette smoking, ionizing radiation, metabolism, etc.) [10,11,12,13]. The main producer of ROS in the cell consists of the nicotinamide adenine dinucleotide phosphate oxidase (NADPH-NOX) enzymes localised in the cell membrane. Further sources of ROS are uncoupled endothelial nitric oxide synthase (eNOS), the endoplasmic reticulum, and the mitochondria [10,11,12,13]. Oxidative stress may induce DNA damage. The most serious type of DNA damage consists of DNA breaks (single strand breaks (SSBs) are less serious, whereas double strand breaks (DSBs) are more serious). DNA damage is quickly repaired through the DNA damage response (DDR) system. When the DDR system is damaged or overloaded, DNA damage remains unrepaired. An abundance of DNA lesions leads to excessive poly-ADP ribose polymerase (PARP1) activation and results in the depletion of protective substances (nicotinamide adenine dinucleotide (NAD+), adenosine-triphosphate (ATP), sirtuins (SIRTs)), leading to irreversible cell necrosis [10,11,12,13]. Unrepaired DNA is shifted from the nucleus to the cytoplasm as a micronucleus. The membrane of the micronucleus is brittle and disintegrates in the cytoplasm. Unrepaired DNA accumulates in the cytoplasm when the autophagic and lysosomal defences of the cytosol are exceeded. There are two main specific cell sensor pathways sensing harmful cytosolic DNA ((1) the absent in melanoma (AIM2) inflammasome and (2) the cyclic GMP-AMP (cGAMP) synthase—stimulator of interferon genes, or cGAS–cGAMP–STING, pathway) that, through known pro-inflammatory mediators, inflammation, fibrosis, and a vascular wall remodelling cascade, lead to hypertension and organ complications [1,40]. *Copyright is not needed, all illustrations are originally created by the author of this text based on known facts*.

In contrast to other CV risk factors, summarized evidence shows consistent increase of ox-DNA damage in HT [26,27,41,42,43,44,45,46,47,48,49,50,51,52,53,54,55,56,57,58,59,60,61,62,63,64,65,66,67,68,69,70,71,72,73,74,75,76,77,78,79,80,81,85,86,87,88,89,90,91,92,93,94,95,96,97,98,99,100,101,102,103,104,105,106,107,108,109,110,111,112,113,114,115,116,117,118,119,120] (Figure 3 and Figure 4). Taking into account the known facts about DNA (the crucial biopolymer for life and health) [40], ox-stress and ox-DNA damage [1,2,10,11,12,13,14,15,16,17,18,19,20,21,22], DDR system involving DDR system overload or damage [10,11,12,13], consequences of DNA damage when stays unrepaired including cytosolic DNA sensing pathways and inflammation [40], authors tried to put together a mosaic and linked it to known data from the genesis of hypertension [1]. The result is the proposed concept of HT genesis innovatively supplemented with oxidative DNA damage (Figure 4), that requires verification by further studies. 

## 5. Conclusions

To the best of our knowledge, this review was the first to summarise data on ox-DNA damage in both HT and other major CV risk factors. Data showing higher levels of DNA damage in HT compared with control conditions are consistent and confirmed by studies under strict experimental conditions. Results related to the other CV risk factors are less clear. These facts support a multifactorial and possibly personalized aetiology of HT. This review highlighted the proven relationship between ox-DNA damage and HT. Therefore, the authors propose to supplement the existing concept of HT genesis by ox-DNA damage—an innovative concept is introduced. This review at least partially fills the gap in evidence regarding HT genesis defined in the current guidelines. Both HT genesis and DNA damage are interesting and complex topics requiring further study. The authors believe that this paper supports the vision that components of the DDR system (e.g., PARP) may present promising therapeutic targets for the management of HT.
